# Smartphone-Based Speech Therapy for Poststroke Dysarthria: Pilot Randomized Controlled Trial Evaluating Efficacy and Feasibility

**DOI:** 10.2196/56417

**Published:** 2024-04-25

**Authors:** Yuyoung Kim, Minjung Kim, Jinwoo Kim, Tae-Jin Song

**Affiliations:** 1 Human Computer Interaction Lab, Graduate Program in Cognitive Science Yonsei University Seoul Republic of Korea; 2 HAII Corporation Seoul Republic of Korea; 3 Department of Neurology Seoul Hospital Ewha Womans University College of Medicine Seoul Republic of Korea

**Keywords:** dysarthria, stroke, smartphone, speech therapy, app, acute and early subacute, feasibility, mobile phone

## Abstract

**Background:**

Dysarthria is a common poststroke speech disorder affecting communication and psychological well-being. Traditional speech therapy is effective but often poses challenges in terms of accessibility and patient adherence. Emerging smartphone-based therapies may offer promising alternatives for the treatment of poststroke dysarthria.

**Objective:**

This study aimed to assess the efficacy and feasibility of smartphone-based speech therapy for improving speech intelligibility in patients with acute and early subacute poststroke dysarthria. This study also explored the impact of the intervention on psychological well-being, user experience, and overall feasibility in a clinical setting.

**Methods:**

Participants were divided into 2 groups for this randomized, evaluator-blinded trial. The intervention group used a smartphone-based speech therapy app for 1 hour per day, 5 days per week, for 4 weeks, with guideline-based standard stroke care. The control group received standard guideline-based stroke care and rehabilitation. Speech intelligibility, psychological well-being, quality of life, and user acceptance were assessed using repeated measures ANOVA.

**Results:**

In this study, 40 patients with poststroke dysarthria were enrolled, 32 of whom completed the trial (16 in each group). The intervention group showed significant improvements in speech intelligibility compared with the control group. This was evidenced by improvements from baseline (*F*_1,30_=34.35; *P*<.001), between-group differences (*F*_1,30_=6.18; *P*=.02), and notable time-by-group interactions (*F*_1,30_=6.91; *P*=.01). Regarding secondary outcomes, the intervention led to improvements in the percentage of correct consonants over time (*F*_1,30_=5.57; *P*=.03). In addition, significant reductions were noted in the severity of dysarthria in the intervention group over time (*F*_1,30_=21.18; *P*<.001), with a pronounced group effect (*F*_1,30_=5.52; *P*=.03) and time-by-group interaction (*F*_1,30_=5.29; *P*=.03). Regarding quality of life, significant improvements were observed as measured by the EQ-5D-3L questionnaire (*F*_1,30_=13.25; *P*<.001) and EQ-VAS (*F*_1,30_=7.74; *P*=.009) over time. The adherence rate to the smartphone-based app was 64%, with over half of the participants completing all the sessions. The usability of the app was rated high (system usability score 80.78). In addition, the intervention group reported increased self-efficacy in using the app compared with the control group (*F*_1,30_=10.81; *P*=.003).

**Conclusions:**

The smartphone-based speech therapy app significantly improved speech intelligibility, articulation, and quality of life in patients with poststroke dysarthria. These findings indicate that smartphone-based speech therapy can be a useful assistant device in the management of poststroke dysarthria, particularly in the acute and early subacute stroke stages.

**Trial Registration:**

ClinicalTrials.gov NCT05146765; https://clinicaltrials.gov/ct2/show/NCT05146765

## Introduction

Stroke is a leading cause of mortality and morbidity worldwide [1]. Approximately 40% of people who had survived a stroke experience disabilities [2,3], and over half of the patients with acute stroke develop motor speech disorders, particularly dysarthria [4]. Poststroke dysarthria results from weakened, slow, or impaired speech production muscles caused by cranial nerve damage [5]. Poststroke dysarthria can cause abnormalities in vocal quality, pace, strength, and volume, ultimately leading to reduced speech intelligibility. Consequently, decreased speech intelligibility may trigger communication problems, impaired social interactions, anxiety, depression, and decreased quality of life [6,7].

Starting speech therapy immediately after a stroke can enhance recovery [8-10]. Evidence indicates that early, consistent, intensive treatment yields significantly better outcomes [11,12]. However, despite the recognized importance of early intervention, there is a notable lack of clinical studies that specifically address poststroke dysarthria, particularly in the early stages of stroke. The lack of evidence underscores the need for further studies. In animal studies, neuroplastic changes after an ischemic stroke have been shown to aid neural recovery. However, the direct applicability of these findings in human patients remains uncertain [13,14]. Therefore, further research is needed to define the benefits and risks of early interventions after stroke [10].

Unfortunately, treatment adherence is negatively affected by the perception that current speech treatments are tedious and repetitive [15]. Furthermore, patients may face restrictions regarding therapeutic resources because speech therapy requires substantial time and effort by clinicians or speech-language pathologists (SLPs) [16]. Approximately one-third of the patients received sufficient speech therapy. Additionally, the amount and frequency of therapy received varies among patients [17].

Digital speech therapy apps may offer significant advancements over traditional approaches [18,19]. They also enhance therapeutic accessibility and patient engagement. Additionally, they deliver effective therapeutic dosages and offer tailored feedback to patients [6]. Most importantly, smartphone-based speech therapy apps offer flexibility and ease of access. This is particularly beneficial for patients with stroke who find clinic visits challenging. In addition, smartphone-based speech therapy apps can reduce time and economic burden [20]. Smartphone-based speech therapy can play a crucial role in increasing therapy intensity. High-intensity practice leads to better outcomes in poststroke dysarthria treatment [5,21]. Smartphone-based speech therapy can be delivered using multimedia resources. This approach enhances patient engagement through repetitive practice. Finally, smartphone-based speech therapy enables patients to practice speech independently by measuring various vocal parameters and providing tailored feedback [22]. Real-time feedback can assist patients in recognizing and correcting inappropriate speech patterns [23,24]. This system can enhance the effectiveness of speech therapy by providing valuable insights and improving motivation. Additionally, such feedback is crucial to enhance patient self-efficacy and promote positive behavioral changes [25].

Our primary aim was to evaluate the effect of smartphone-based speech therapy on speech intelligibility, particularly in patients with poststroke dysarthria in the acute and early subacute stroke stages. Additionally, we focused on articulation function, dysarthria severity, and psychological well-being, including depression, anxiety, and quality of life. This study also assessed the feasibility of the trial by examining the adherence, recruitment, and dropout rates. Furthermore, we evaluated the usability and self-efficacy of the app experienced by the participants. This study aimed to explore the efficacy of early intervention and assess how digital tools can enhance speech therapy outcomes in patients with poststroke dysarthria.

## Methods

### Study Design

This was a prospective, randomized, evaluator-blinded trial study. Participants were allocated to intervention and control groups. They were recruited from 2 stroke centers in South Korea: Ewha Womans University Seoul Hospital and Mokdong Hospital. The trial was registered at ClinicalTrials.gov (NCT05146765).

The participants were screened for eligibility and randomly allocated to the intervention or control groups. Demographic and clinical characteristics were recorded, and a detailed baseline assessment of poststroke dysarthria was conducted. After 4 weeks, the participants underwent a postevaluation to reassess their condition and measure the efficacy of the intervention. The trial was designed according to the CONSORT-EHEALTH (Consolidated Standards of Reporting Trials of Electronic and Mobile Health Applications and Online Telehealth) checklist (Multimedia Appendix 1).

### Ethical Considerations

In adherence to our commitment to ethical research standards, we observed several vital considerations throughout this study. Our adherence to these ethical principles was fundamental to ensuring all participants’ dignity, rights, safety, and well-being. Upon receiving ethics approval from the Ewha Womans University Seoul Hospital Institutional Review Board (approval SEUMC 2021-12-011), we ensured that all research procedures strictly adhered to the guidelines outlined in the Declaration of Helsinki [26]. Before participating, participants identified as neurologically stable and survived a stroke in the acute and early subacute stages received detailed information about the study’s goals, procedures, and potential benefits and risks. Each participant provided written informed consent to affirm their voluntary participation and understanding of the study. This consent process was necessary to ensure participants were fully informed and their autonomy respected. Next, strict data protection measures were implemented to safeguard our participants’ privacy and confidentiality. All collected data were anonymized throughout the research process to preserve participants’ privacy. We offered participants a monetary compensation of ₩50,000 (US $40) for their involvement in the study, which amounts to ₩25,000 (US $20) per visit. This compensation was offered as a token of appreciation for their valuable contribution to our research and to acknowledge the personal investment each participant made by dedicating their time to our study.

### Participants

A principal investigator (TJS), specializing in stroke, screened and enrolled the eligible participants. The inclusion criteria were as follows: (1) patients diagnosed with dysarthria by a stroke specialist [27], (2) patients who are neurologically stable, (3) first-time patients with stroke, (4) patients who are in the acute or early subacute phase of stroke defined as having experienced their initial stroke event within the past 1 month, (5) patients with sufficient cognitive abilities to use a smartphone-based speech therapy app (Mini-Mental State Examination score ≥26) [28], and (6) patients with adequate vision [29], hearing [30], communication skills, and motor skills [27]. The exclusion criteria were as follows: (1) coexisting language disorders (eg, aphasia) or neurological disorders (eg, dementia, Pick disease, Huntington disease, Parkinson disease, or Parkinsonism) that could influence dysarthria, (2) history of severe mental disorders (eg, depression, schizophrenia, alcohol addiction, or drug addiction), (3) inability to use or access smartphone technology, (4) illiteracy, and (5) inability to communicate in Korean, the primary language of the study location.

### Randomization and Masking

An independent researcher managed the randomization. A computerized system with permuted block sizes of 2 and 4 was used to ensure a balanced and unpredictable group distribution [31]. The block sizes were disclosed to the participants or researchers at the end of the trial to ensure randomization.

Given the intervention’s interactive nature, it was impossible to blind the participants to their group assignments [32]. However, independent evaluators and those not involved in the treatment process were blinded to the group allocation to minimize potential bias. This masking was crucial to maintain the integrity of the assessment. To preserve the integrity of the blinded assessment, participants were instructed not to disclose any intervention-related details during the evaluation sessions.

### Intervention

#### Intervention Group

Participants in the intervention group received a smartphone-based speech therapy app and standard guideline-based stroke care. This app allowed participants to achieve speech therapy independently without the support of caregivers or therapists. The participants were instructed to use the app for 1 hour daily for at least 5 days per week for 4 consecutive weeks. The intervention could be completed in a single session or distributed across multiple daily sessions.

The app was tailored for older adults with poststroke dysarthria and optimized for users facing age-related challenges [33]. The interface was designed to minimize unintentional interactions for participants with motor impairments. Intuitive design elements, such as sequential tabs and text-labeled buttons, were included to enhance usability for older adults [34]. Moreover, button size and spacing were adjusted to facilitate ease of use and reduce inadvertent presses.

The app provided 6 components of speech exercises for 1 day based on established behavioral therapies [5,10]. These included oro-motor exercises, sustained sound, pitch variation, velopharyngeal closure, reading practice, and syllable repetition (Table 1). The primary goal of these exercises was to improve overall speech intelligibility and enhance articulation.

**Figure 1:**
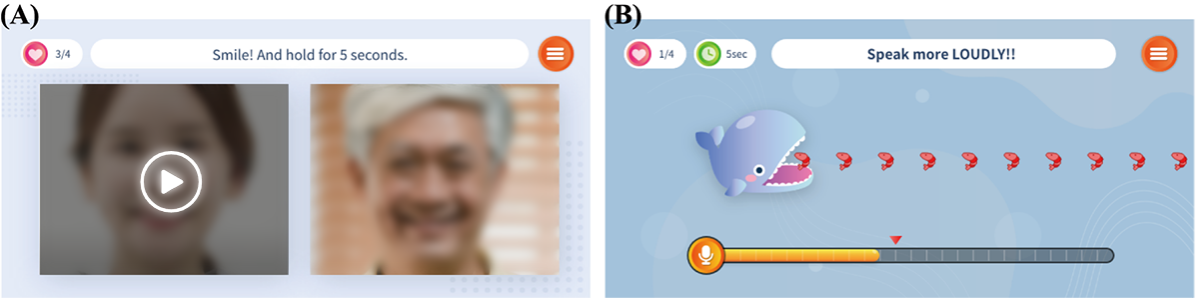
Two components of the smartphone-based speech therapy app. (A) Oro-motor exercise videos for specific muscle stretches with camera mirroring. (B) Speech exercises (sustaining sound) with real-time feedback to improve articulation.

Speech exercises such as sustaining sounds, repeating syllables, and reading provided real-time auditory and visual feedback. Real-time feedback was provided throughout the sessions to promote attention and self-awareness during speech therapy. Pronunciations and speech signals were transmitted during speech exercises. Our engine analyzed the speech parameters and provided feedback. The feedback results were displayed on the participants’ devices. For instance, the “sustaining sound” task required participants to sustain a vowel sound, such as /ah/, for 5-15 seconds. Subsequently, real-time feedback on the loudness, sound length, and pitch was provided. Participants could address speech errors through insights gained from the feedback (eg, “Speak more loudly!” in Figure 1B).

**Figure 2:**
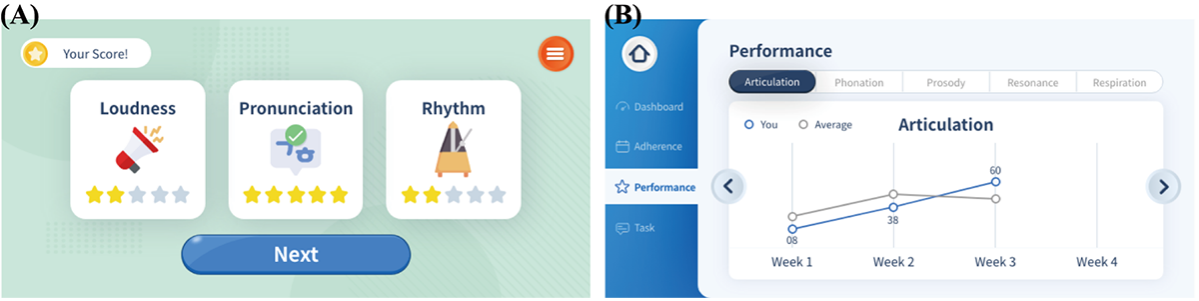
Treatment results in the smartphone-based speech therapy app. (A) Session summary with scores based on speech exercise performance. (B) Cumulative analysis presenting daily and weekly or monthly speech outcome scores.

The treatment results are presented in 2 formats as shown in Figure 2. First, a summary of each therapy session focused on speech outcomes, including pronunciation accuracy, loudness, and pitch. The participants understood these outcomes better through voice- and text-guided interactions. After the exercise, they listened to their recorded voices and provided feedback. This feedback helped them assess their progress (Figure 2A). Second, the app provided cumulative analysis. The analysis included the daily treatment results, weekly and monthly progress, and speech outcome scores (Figure 2B).

The app automatically logged all the results in a database. The researchers could access these results using a data-logging system. Researchers monitored the participants’ adherence to the intervention and offered coaching for lapses or technical issues. The researchers evaluated the app use every evening to monitor participants’ adherence. If reduced adherence was observed, the researchers contacted the participants the following day via phone call or SMS text message to encourage therapeutic engagement. The participants were encouraged to report any app-related issues, which were promptly addressed by the researchers.

**Table 1:** Procedure for each speech exercise.

Exercise	Procedure	Outcome monitoring
Oro-motor exercise	Oro-motor exercises were designed to relax oral muscles and actively engage them. These exercises improved muscle coordination and strength. A 5-minute instructional video guided users through specific exercises targeting the lips, tongue, cheeks, and jaw. Participants could use a camera to mirror these actions for real-time comparison with the guide in the video.	Compliance rate
Sustaining sound	Sustaining a vowel sound was designed to improve vocal control and strength. Participants practiced holding a vowel sound clearly and loudly for 5 to 15 seconds. They received real-time visual feedback on pitch and duration to help maintain a steady pitch and extend the sound. This approach promoted effective and precise vocal training.	Loudness, pitch, and length
Pitch variation	Pitch variation exercises improved speech intonation and expressiveness. Participants practiced raising and lowering vocal pitch with “do-re-mi” exercises. The app provided visual feedback for these pitch changes. This feedback allowed participants to adjust their pitch with precision in real time. Participants could enhance the efficiency and effectiveness of the exercise by ensuring they achieve the correct pitch.	Length, intonation, and pitch
Velopharyngeal closure	The velopharyngeal closure exercise focused on improving clear speech articulation. This exercise involved practicing words that require the closure of the velopharynx. Participants started by blocking the velopharynx at the beginning of each word. They held this block for 5 seconds to build muscle strength and then released the breath on the following syllable. The app supported this process by showing anatomical animations. These animations aided users in comprehending muscle tension and the timing for its application and release. Emphasis was placed on maintaining a 5-second hold and correctly timing the exhalation.	Loudness, pronunciation accuracy, and breath-holding time
Reading	Reading exercises were structured to improve articulation accuracy and speech intelligibility. They began with single words and then progressed to sentences and paragraphs. This repeated practice enhanced speech precision. Participants were encouraged to read aloud slowly and clearly. The app initially guided pronunciations to demonstrate how to read. It also provided visual cues for participants to slowly read along. Participants practiced reading with the provided material at a steady pace.	Loudness, rhythmic accuracy, and speech rate
Repeating syllables	The repeating syllables exercise was designed to improve articulation and control over speech rate. Participants were instructed to repeat specific syllables, such as /pa/, in sync with a given rhythm. The exercise started slowly and gradually sped up the repetition speed. Visual rhythm cues were provided to help participants maintain the appropriate speed. This method enhanced speech fluidity, precision, articulation, and rhythm control.	Loudness, pronunciation accuracy, and rhythmic accuracy

#### Control Group

Participants in the control group received standard stroke care for 4 weeks. Standard stroke care includes medical treatment, routine stroke therapy, and rehabilitative exercises, as outlined in the basic guidelines [35,36]. This care encompassed the conventional speech treatment recommended in standard protocols, such as vocal and articulation exercises. Care was provided by clinicians and SLPs who adhered to the conventional stroke therapy methods. Treatment was tailored to each participant’s clinical needs, established through a collaborative agreement between clinicians and participants, and modified to reflect their progress. Additionally, after the 4-week study period, participants in the control group were allowed to use a smartphone-based speech therapy app.

### Outcome Measures

Assessments were conducted at 2 time points: at baseline and then immediately after the 4-week intervention period.

#### Primary Outcome: Speech Intelligibility

The primary outcome of this study was a change in speech intelligibility. To evaluate speech intelligibility, participants were asked to read the “Gaeul” passage, a standardized tool used in Korean paragraph reading tests for speakers with motor speech disorders, developed by Kim [37]. This passage consists of 369 syllables representing the frequency of occurrence of Korean vowels and consonants.

Participants were instructed to read the passage aloud at their natural pitch and loudness. Recordings were made using a high-quality digital recorder (Sony ICD-UX560F) positioned 30 cm from the participants in a quiet room. The evaluation was carried out in an environment free from noise, which ensured that the conditions were consistent for every assessment [38]. Participants were seated close to the evaluator to ensure optimal sound quality. The primary SLP evaluator conducted the assessment in the room during the recording. Subsequently, experienced SLPs, who were blinded to the participants’ group allocation, listened to each recording and assessed the speech intelligibility. All 3 SLPs who conducted the assessment possessed over 6 years of clinical experience, specialized in poststroke dysarthria, and held certifications in Korean speech-language pathology. Additionally, they had experienced specialized training in poststroke dysarthria. Speech intelligibility was rated on a scale ranging from 0 (intelligible, can be understood without difficulty) to 6 (unintelligible, cannot be understood at all) [39]. The other 2 evaluators assessed speech intelligibility based on the recorded audio. The average score from the 3 SLPs was used to determine each participant’s final speech intelligibility score.

#### Secondary Outcomes

Secondary outcomes were measured to assess factors related to dysarthria and psychological well-being. First, the Urimal Test of Articulation and Phonology 2 (U-TAP2) was used [40]. This measurement was used to identify the percentage of consonants correct for detecting articulation anomalies [41]. Participants were asked to read 30 words from U-TAP2 in a quiet room. The SLPs then recorded these readings and calculated the percentage of consonants correct by marking misarticulated consonants (94 in total) and converting them into a percentage score.

Stroke-related neurological deficits were measured using the National Institute of Health Stroke Scale [27], with a specific focus on components related to dysarthria. Stroke specialists quickly evaluated the severity of dysarthric speech. As the participants spoke specific words, the severity was rated on a 3-point scale: 0=normal, 1=mild to moderate, and 2=severe. This assessment was conducted by a seasoned neurologist with over 20 years of experience in stroke specialization and certified in the Korean National Institute of Health Stroke Scale.

Finally, participants’ psychological well-being was measured using self-reported questionnaires. The Patient Health Questionnaire-9 [42,43] and the Generalized Anxiety Disorder 7-Item Scale [44,45] were used to evaluate depressive and anxiety symptoms. Furthermore, the EQ-5D-3L questionnaire [46] was used to assess the participants’ quality of life across 5 different areas: their ability to move around, care for themselves, perform their usual activities, levels of pain or discomfort, and mood. To assign specific values to these quality-of-life measures, we applied weights based on the preferences of the South Korean population. These weights were calculated using the time trade-off method and scores from a visual analog scale [47].

#### Feasibility and User Acceptance

Feasibility was assessed based on several aspects. The participant recruitment rates were documented to reflect the level of engagement. Adherence to the intervention was evaluated by tracking the completion rates of the prescribed speech therapy sessions within the app, the frequency of app use, and the average duration of each session. These data, which were transmitted to a dedicated web system, allowed for a detailed analysis of adherence. Potential adverse events and safety concerns were continuously monitored. Any reported issues with app use or challenges faced by the participants were investigated by analyzing the app’s log data.

The usability and acceptance of smartphone-based speech therapy apps were measured using 2 surveys: the System Usability Scale (SUS) [48] and the Modified Computer Self-Efficacy Scale (mCSES) [49]. The usability of the app was evaluated using a 10-item, 5-point Likert scale that measured effectiveness, efficiency, and satisfaction. The mCSES was used to gauge participants’ confidence in using the new technology, especially tailored for older patients and those with disabilities.

### Statistical Analysis

Power analysis focused on measuring the changes in speech intelligibility. We initially calculated that 32 participants were required to achieve 80% power [50] to detect a moderate effect size of 0.29 [51] with a significance level set at .02. However, we aimed to enroll 8 more participants to account for an anticipated dropout rate of 25%. Therefore, our goal was to recruit 40 participants with 20 participants per group [52].

Descriptive statistics (mean, SD, and percentage) were used to summarize the clinical and demographic characteristics of the participants. To ensure homogeneity between the intervention and control groups, a 2-tailed independent sample *t* test was conducted for continuous variables, whereas a chi-square test was used for categorical variables. Following the intention-to-treat principle, repeated measures ANOVA was applied to detect changes in outcome measures between and within groups. This analysis incorporated fixed effects for time, group, and time-by-group interactions, with measures taken at baseline and 4 weeks after the intervention. All analyses were performed using SPSS (version 27.0; IBM Corp). Statistical significance was set at *P*<.05 and was considered statistically significant.

### Data Management

All data were encrypted to ensure privacy. After encryption, the system was securely transmitted to a dedicated web system. This process maintained the confidentiality and safety of the data. Real-time data such as app use frequency, session duration, and speech performance metrics are necessary for monitoring therapeutic progress and adapting the intervention as needed. Our research team used proactive measures to ensure consistent participation. For instance, reduced adherence to the app triggered alerts, which prompted our team to reconnect with the participants to understand and address their concerns. While participants could withdraw from the study at any time, the research team reserved the right to exclude those who required immediate medical attention for reasons that were not limited to the study parameters.

## Results

### Participants

We recruited 129 patients with acute to early subacute cerebral infarction between January 18, 2022, and May 31, 2022. These patients were screened based on the eligibility criteria. Of these, 81 patients exhibited symptoms of dysarthria. During the screening process, 14 patients were excluded due to coexisting aphasia, 10 due to psychological problems or medication, 11 due to dementia or cognitive dysfunction, 3 due to inability to use or access smartphone technology, and 3 due to visual or hearing impairment. Finally, 40 participants were enrolled.

**Figure 3:**
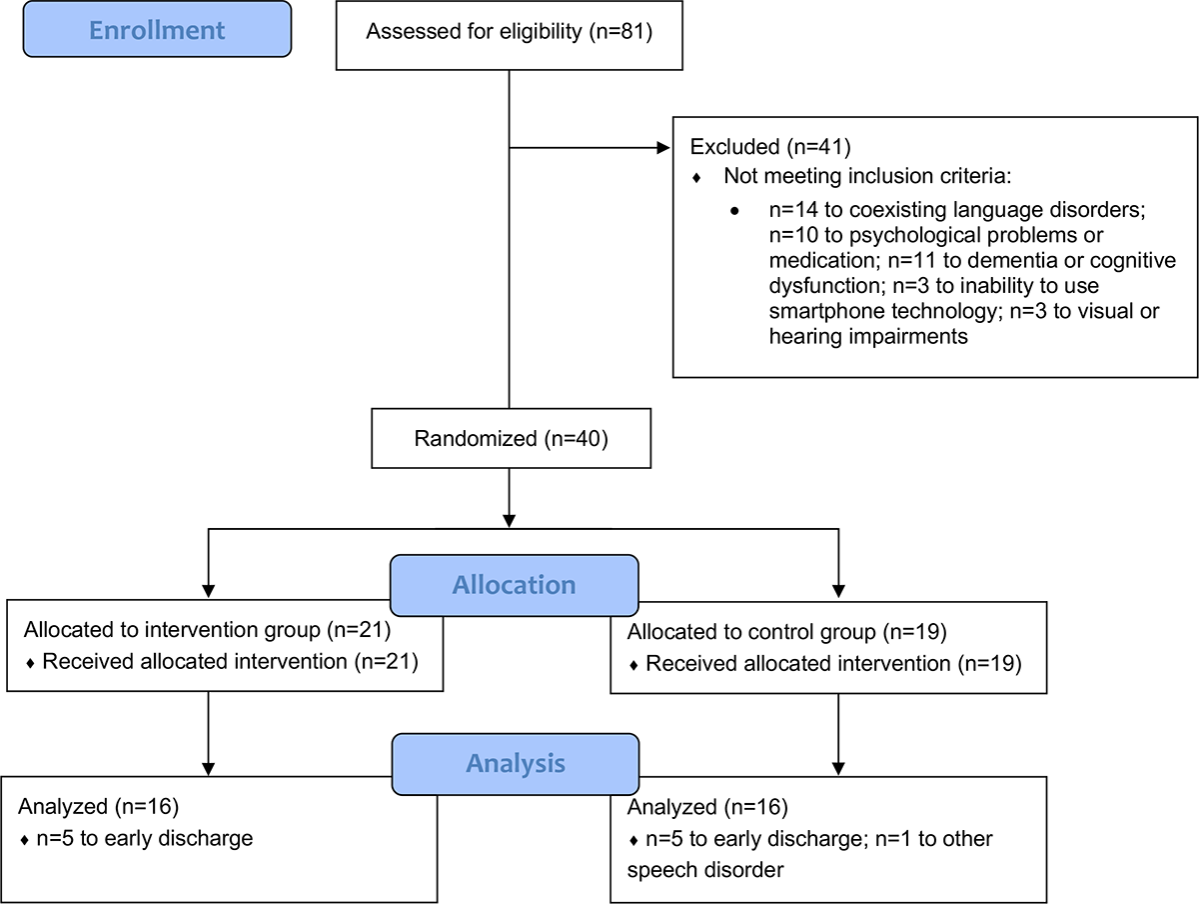
CONSORT (Consolidated Standards of Reporting Trials) flow diagram.

The 40 participants were randomized into 2 study groups, as shown in Figure 3. We excluded 7 participants who could not complete the study for personal reasons: 5 in the intervention group and 2 in the control group. Additionally, 1 participant in the control group was excluded because of another speech disorder, apraxia. The final analysis included 32 participants (16 each in the treatment and control groups).

Table 2 presents the baseline characteristics of the participants. Chi-square and independent 2-tailed *t* tests revealed no significant differences between the 2 study groups. Among the 32 participants, 25 were male and 7 were female, with a mean age of 65.25 (SD 12.97; treatment group: mean 60.44, SD 11.94 and control group: mean 70.06, SD 12.47) years. All the participants were in the acute and early subacute phases of poststroke dysarthria. The treatment group participants were observed for an average of 7.06 (SD 3.66) days after stroke. In contrast, the control group participants were assessed on an average of 7.88 (SD 6.45) days after stroke.

**Table 2:** Baseline demographics and clinical characteristics of participants.

Participants characteristics			Intervention (n=16)		Control (n=16)		Total (n=32)		Chi-square (*df*)	*P* value	
**Sex, n (%)**									1.6 (1)		.20
	Male	14 (44)		11 (34)		25 (78)					
	Female	2 (6)		5 (16)		7 (22)					
Age (years), mean (SD)			60.44 (11.94)		70.06 (12.47)		65.25 (12.97)		26.0 (21)	.21	
**Type of stroke, n (%)**											
	Cerebral infarction	16 (50)		15 (47)		31 (97)		N/A^a^		N/A	
	Transient ischemic attack	0 (0)		1 (3)		1 (3)		N/A		N/A	
Duration after stroke (days), mean (SD)			7.06 (3.66)		7.88 (6.45)		7.47 (5.17)		8.7 (12)	.73	
**Speech ability, mean (SD)**											
	Speech intelligibility	1.56 (0.89)		2.31 (1.30)		1.94 (1.16)		4.3 (4)		.37	
	U-TAP2^b^ (%)	95.67 (3.33)		91.41 (7.34)		93.54 (6.01)		9.4 (12)		.47	
	NIHSS^c^ dysarthria	1.00 (0.00)		1.06 (0.25)		1.03 (0.18)		1.0 (1)		.31	
**Psychological well-being, mean (SD)**											
	PHQ-9^d^	5.88 (5.26)		12.13 (7.07)		9.00 (6.90)		18.0 (17)		.39	
	GAD-7^e^	5.25 (5.25)		7.69 (6.89)		6.47 (6.15)		11.5 (12)		.48	
	EQ-5D-3L	0.73 (0.28)		0.57 (0.30)		0.65 (0.30)		19.6 (18)		.36	
	EQ-VAS	69.88 (15.24)		52.19 (28.28)		61.03 (24.09)		19.5 (11)		.04	
**Feasibility, mean (SD)**											
	mCSES^f^	66.50 (33.28)		40.56 (26.48)		53.53 (32.38)		26.0 (21)		.21	

^a^N/A: not applicable.

^b^U-TAP2: Urimal Test of Articulation and Phonology 2.

^c^NIHSS: National Institute of Health Stroke Scale.

^d^PHQ-9: Patient Health Questionnaire-9.

^e^GAD-7: Generalized Anxiety Disorder 7-Item Scale.

^f^mCSES: Modified Computer Self-Efficacy Scale.

### Primary Outcome

During the baseline assessment, none of the participants were rated as 0=completely understandable or 6=completely unintelligible. Of the total 32 participants, 16 had a rating of 1, indicating slight difficulties in speech intelligibility. Another 8 participants had a rating of 2, demonstrating mild dysarthria. A range of speech intelligibility issues was observed: 5 participants had a rating of 3, which indicated moderate dysarthria; and 2 participants had a rating of 4, which suggested more severe difficulties. Only 1 participant had a rating of 5, which indicated they were close to being unintelligible.

Repeated measures ANOVA was conducted to assess the impact of time, group, and time-by-group interactions on speech intelligibility. The results revealed a significant effect of time (*F*_1,30_=34.35; *P*<.001). This finding indicated that there were significant changes in speech intelligibility between baseline and 4 weeks after the intervention. The mean speech intelligibility score in the intervention group improved from 1.56 (SD 0.89) at baseline to 0.69 (SD 1.09) after intervention. Additionally, a significant group effect was observed (*F*_1,30_=6.18; *P*=.02). This analysis suggested significant differences in speech intelligibility between the treatment and control groups. Furthermore, the interaction effect between time and group was also significant (*F*_1,30_=6.91; *P*=.01), which indicates that the changes in speech intelligibility over time varied significantly between the groups.

### Secondary Outcomes

The intervention group demonstrated notable improvements in secondary outcomes compared with the control group after intervention (Table 3).

**Table 3:** Repeated measures ANOVA of outcome scores.

Variables			Outcome scores, mean (SD)			Time				Group					Time×group		
			Intervention		Control	*F* test (*df*=1)		*P* value		*F* test (*df*=1)		*P* value		*F* test (*df*=1)			*P* value
**Speech intelligibility**						34.35		<.001		6.18		.02		6.91			.01
	Baseline	1.56 (0.89)		2.31 (1.30)													
	Postintervention	0.69 (1.09)		1.98 (1.43)													
**U-TAP2^a^ (%)**						5.57		.03		3.52		.07		4.13			.05
	Baseline	95.67 (3.33)		91.41 (7.34)													
	Postintervention	95.88 (2.93)		94.22 (4.38)													
**NIHSS^b^ dysarthria**						21.18		<.001		5.52		.03		5.29			.03
	Baseline	1.00 (0.00)		1.06 (0.25)													
	Postintervention	0.44 (0.51)		0.87 (0.50)													
**PHQ-9^c^**						1.42		.24		8.33		.007		0.66			.42
	Baseline	5.88 (5.26)		12.13 (7.07)													
	Postintervention	5.38 (4.10)		9.50 (7.98)													
**GAD-7^d^**						2.09		.16		2.15		.15		0.13			.91
	Baseline	5.25 (5.25)		7.69 (6.87)													
	Postintervention	3.56 (4.87)		6.25 (6.05)													
**EQ-5D-3L**						13.25		<.001		3.64		.07		0.76			.79
	Baseline	0.73 (0.28)		0.57 (0.30)													
	Postintervention	0.87 (0.12)		0.74 (0.25)													
**EQ-VAS**						7.74		.009		6.06		.02		0.15			.70
	Baseline	69.88 (15.24)		52.19 (28.28)													
	Postintervention	79.06 (14.17)		64.31 (25.10)													
**mCSES^e^**						2.99		.09		10.81		.003		0.97			.33
	Baseline	66.50 (33.28)		40.56 (26.48)													
	Postintervention	77.88 (25.01)		43.69 (28.38)													

^a^U-TAP2: Urimal Test of Articulation and Phonology 2.

^b^NIHSS: National Institute of Health Stroke Scale.

^c^PHQ-9: Patient Health Questionnaire-9.

^d^GAD-7: Generalized Anxiety Disorder 7-Item Scale.

^e^mCSES: Modified Computer Self-Efficacy Scale.

First, the percentage of correct consonants measured by the U-TAP2 showed a significant time effect (*F*_1,30_=5.57; *P*=.03) compared to the change between baseline and 4 weeks after the intervention. However, the group effect (*F*_1,30_=3.52; *P*=.07) and time-by-group interaction (*F*_1,30_=4.13; *P*=.05) were not statistically significant.

Second, significant findings emerged from the assessment of the severity of poststroke dysarthria. The time effect was significant (*F*_1,30_=2.21; *P*≤.001). This highlights a notable improvement in the severity over 4 weeks. Furthermore, a significant group effect (*F*_1,30_=5.52; *P*=.03) indicated differences in severity between the treatment and control groups. Most importantly, the significant time-by-group interaction (*F*_1,30_=5.29; *P*=.03) suggests that the groups experienced different trajectories of severity over time.

Third, no significant benefits were observed for depression or anxiety. For depression, as measured by the Patient Health Questionnaire-9, there was no significant time effect (*F*_1,30_=1.42; *P*=.24), and the time-by-group interaction was also not significant (*F*_1,30_=0.66; *P*=.42). However, a significant group effect was observed (*F*_1,30_=8.33; *P*=.007). In terms of anxiety levels, as assessed by the Generalized Anxiety Disorder 7-Item Scale, no significant effects were found for time (*F*_1,30_=2.09; *P*=.16; group: *F*_1,30_=2.15; *P*=.15; or time-by-group interaction: *F*_1,30_=0.13; *P*=.91).

Finally, a significant time effect was noted for the overall quality of life measured by the EQ-5D-3L (*F*_1,30_=13.25; *P*≤.001). No significant effects were observed for group (*F*_1,30_=3.64; *P*=.07) or time-by-group interactions (*F*_1,30_=0.76; *P*=.79). In addition, the EQ-VAS scores showed a significant time effect (*F*_1,30_=7.74; *P*=.009) and group effect (*F*_1,30_=6.06; *P*=.02). However, there was no significant time-by-group interaction (*F*_1,30_=0.15; *P*=.70).

### Feasibility

We met our recruitment goal by successfully enrolling 40 participants during the study period. The final assessment completion rate was 80%. Regarding adherence, 64% (n=20) of participants in the intervention group consistently used the smartphone-based speech therapy app throughout the designated period. More than 51% (n=16) of the participants completed the prescribed sessions.

System usability was considered excellent, as measured by the mean SUS score of 80.78 (SD 16.27). Concerning self-efficacy, measured by the mCSES, the intervention group had a substantial group effect (*F*_1,30_=10.81; *P*=.003), but there were no significant changes over time (*F*_1,30_=2.99; *P*=.09) or in the time-by-group interaction (*F*_1,30_=0.97; *P*=.33). No significant adverse events were observed during the study period.

## Discussion

### Principal Findings

Despite its significant impact on communication and psychosocial well-being, poststroke dysarthria remains underresearched. In particular, there is a lack of evidence on poststroke dysarthria interventions, highlighting the urgent need for more comprehensive research [53]. Understanding the prognosis of speech therapy in the critical initial months after stroke is vital because early intervention can hasten recovery [9]. Unfortunately, there is a knowledge gap in the evidence regarding poststroke dysarthria during the acute and early subacute phases [54]. Our trial findings provide evidence of the efficacy of smartphone-based speech therapy in the treatment of poststroke dysarthria.

In this study, participants experienced significant improvements in speech intelligibility and articulation after 4 weeks of using the smartphone-based speech therapy app compared to those receiving standard stroke care. This intervention was effective in several ways. It showed the potential for reducing the severity of dysarthria. It also helped alleviate depression and improve the quality of life of the participants. Consistent with prior studies, these results underscore the reliability of smartphone-based interventions [55,56].

The efficacy of traditional behavioral speech therapy has been proven in the chronic phase; however, studies on patients with acute and early subacute strokes are limited. Prior studies have shown encouraging results for behavioral speech therapy such as breathing exercises, nonspeech oro-motor exercises, and Lee Silverman Voice Treatment for the chronic poststroke phase [57]. One study used the Lee Silverman Voice Treatment that focuses on high phonatory effort and reading exercises [58]. This study showed promising results in a small group of 4 individuals who have survived a stroke with dysarthria for 9 months. According to another study, repetitive speech therapy had a positive effect on patients with stroke for at least 6 months [59].

Our study expands traditional behavioral speech therapy into a digital format using a smartphone-based app [58-61]. This approach overcomes the limitations of traditional methods by offering more accessible, engaging, and cost-effective speech therapy that enables self-management [62-65]. Patients can perform various speech exercises at home. Home-based treatment reduces the need for frequent clinical visits and reduces expenses [66,67]. Moreover, the app provides uninterrupted therapy sessions, even during the COVID-19 pandemic. This serves as a reliable alternative to clinical treatment [68].

Patients with poststroke dysarthria also commonly experience adverse psychological effects [6,7]. Previous studies focusing on speech therapy in participants with poststroke aphasia have demonstrated improvements in depression [69], anxiety [70], and quality of life [71]. However, specific evidence for poststroke dysarthria remains limited. Although we observed a significant decrease in depressive symptoms, no significant changes in anxiety levels were observed. Notably, the EQ-5D-3L and EQ-VAS scores indicated a substantial improvement in quality of life over time and a positive effect of the intervention. However, the lack of significant group differences in these scores suggests that improvements in quality of life were not solely attributable to the intervention. This divergence in findings highlights the complexity of assessing the full effect of speech therapy interventions on psychological well-being. Due to the significant impact of psychological well-being deterioration in patients with poststroke dysarthria, cognitive behavioral therapy should also be considered as a potential treatment [72]. Since this study is primarily focused on speech intelligibility, it may not have fully captured the broader impact of speech therapy on psychological well-being. Given these findings, there is a clear need for further research with larger sample sizes to provide a better understanding of the benefits of speech therapy interventions on the psychological well-being of patients with poststroke dysarthria. This can help develop effective treatment strategies, specifically in the areas of speech and psychological well-being.

Meanwhile, the average SUS score of 80.78 (SD 16.27) signifies excellent usability, which indicates that the participants found the app user-friendly and efficient. Participants also noted increased self-efficacy in app use compared with before treatment. These results suggest that the app helped overcome apprehensions about using the technology, particularly among older users. This increased system feasibility is a promising sign of active participation in therapy.

However, the treatment adherence was lower than expected. Notably, measuring adherence was challenging because of variable internet connectivity among the participants. Due to low-specification phones or unstable home internet connections, many participants, especially older users, experienced frequent internet disconnections. These challenges hindered the proper storage of log data, which may have led to inaccuracies in adherence measurements. Our app includes features, such as progress graphs and feedback, to address adherence-related issues and encourage self-monitoring [12]. Although these features are standard in health apps and are crucial for self-therapy, they have limited long-term effectiveness [73,74]. This limitation is particularly relevant for older adults who are unfamiliar with digital devices [75,76]. Given these challenges, future research should focus on improving adherence to therapy and making it more accessible to diverse patient groups. Including more subjects and a broader range of variables could enhance our understanding of how digital interventions can be most effectively used in poststroke care. Regarding home therapies, various factors, such as the patient’s social context and home environment, can affect the treatment effectiveness. For example, providing an admin system to monitor and control patient performance data is recommended. This would allow clinicians or family caregivers to remotely track adherence and performance and address potential issues arising from the lack of face-to-face interactions. This could help older adults maintain adherence and maximize the therapeutic effects of treatment [77].

### Limitations

This study has several limitations. First, even as a pilot trial, this study included a small number of participants. Additionally, there was a gender imbalance with a significantly higher number of male participants. Future studies should aim for larger sample sizes and consider recruitment from multiple centers to improve the feasibility and generalizability.

Second, this study focused only on patients with poststroke dysarthria in the acute and early subacute stages. However, dysarthria affects patients in both the acute and chronic stages of stroke. To validate the effectiveness of the intervention across diverse patient profiles, future research should include a broader range of patients with stroke and consider the onset period and severity of dysarthria. Additionally, this study only recruited patients in the acute and early subacute stages of joint impairment after stroke, which may have resulted in the exclusion of patients with severe joint impairment. These selection criteria may have influenced the observed effects of smartphone-based speech therapy. In future studies, it would be beneficial to include participants with varying degrees of dysarthria to understand better the efficacy of this therapy across a spectrum of severity. A more detailed analysis, which may include secondary assessments, can be carried out to evaluate the therapy’s efficacy in addressing speech impairments of varying severity. This approach would enable a deeper understanding of the therapy’s applicability to a broader range of dysarthria cases after stroke.

Third, regarding the measurement of consonant accuracy using U-TAP2 at the word level, we recognize that this approach has limitations, particularly in adult poststroke dysarthria. While U-TAP2 is extensively used to assess articulatory precision in Korean children with developmental articulation disorders, its application is limited [40]. When measuring speech intelligibility in adults with poststroke dysarthria, particularly in continuous speech, U-TAP2 may not fully capture all the complexities. This tool needs to be equipped to grasp the full range of speech intelligibility challenges this adult population faces. Specifically, this method may overlook critical aspects of speech, such as rhythm, prosody, and coarticulation effects, which are essential for understanding overall speech severity. The choice of U-TAP2 was influenced by the absence of standardized assessment tools for adult poststroke dysarthria in the Korean clinical environment. However, we acknowledge that future research should explore more comprehensive tools like the Frenchay Dysarthria Assessment to analyze the various influencing factors of dysarthria more thoroughly [78].

Finally, the smartphone-based speech therapy app used in this study was developed in Korean. Future research should aim to create multilingual versions of the app. Studying multilingual versions would enable researchers to assess their effectiveness across different nationalities and broaden their reach.

### Conclusions

This study emphasized the importance of digital speech therapy in the treatment of poststroke dysarthria. Smartphone apps designed for speech therapy can be used alongside traditional speech therapies and have shown promising results in improving speech outcomes and the overall quality of life. Our findings provide encouraging evidence for the integration of these apps into existing treatment plans. However, more extensive and comprehensive studies are needed to fully understand the impact of digital speech therapy and optimize its use in treating poststroke dysarthria.

## Appendix

Multimedia Appendix 1 CONSORT-EHEALTH (Consolidated Standards of Reporting Trials of Electronic and Mobile Health Applications and Online Telehealth) V 1.6.1 checklist.

## Abbreviations

CONSORT-EHEALTH: Consolidated Standards of Reporting Trials of Electronic and Mobile Health Applications and Online Telehealth
mCSES: Modified Computer Self-Efficacy Scale
SLP: speech-language pathologist
SUS: System Usability Scale
U-TAP2: Urimal Test of Articulation and Phonology 2
